# Effect of Water Invasion on Outburst Predictive Index of Low Rank Coals in Dalong Mine

**DOI:** 10.1371/journal.pone.0132355

**Published:** 2015-07-10

**Authors:** Jingyu Jiang, Yuanping Cheng, Junhui Mou, Kan Jin, Jie Cui

**Affiliations:** 1 Key Laboratory of Gas and Fire Control for Coal Mine, Faculty of Safety Engineering, China University of Mining & Technology, Xuzhou, 221116, China; 2 National Engineering Research Center for Coal & Gas Control, China University of Mining & Technology, Xuzhou, 221116, China; 3 State Key Laboratory of Coal Mine Disaster Dynamics and Control, Chongqing University, Chongqing, 400044, China; China University of Mining and Technology, CHINA

## Abstract

To improve the coal permeability and outburst prevention, coal seam water injection and a series of outburst prevention measures were tested in outburst coal mines. These methods have become important technologies used for coal and gas outburst prevention and control by increasing the external moisture of coal or decreasing the stress of coal seam and changing the coal pore structure and gas desorption speed. In addition, techniques have had a significant impact on the gas extraction and outburst prevention indicators of coal seams. Globally, low rank coals reservoirs account for nearly half of hidden coal reserves and the most obvious feature of low rank coal is the high natural moisture content. Moisture will restrain the gas desorption and will affect the gas extraction and accuracy of the outburst prediction of coals. To study the influence of injected water on methane desorption dynamic characteristics and the outburst predictive index of coal, coal samples were collected from the Dalong Mine. The methane adsorption/desorption test was conducted on coal samples under conditions of different injected water contents. Selective analysis assessed the variations of the gas desorption quantities and the outburst prediction index (coal cutting desorption index). Adsorption tests indicated that the Langmuir volume of the Dalong coal sample is ~40.26 m^3^/t, indicating a strong gas adsorption ability. With the increase of injected water content, the gas desorption amount of the coal samples decreased under the same pressure and temperature. Higher moisture content lowered the accumulation desorption quantity after 120 minutes. The gas desorption volumes and moisture content conformed to a logarithmic relationship. After moisture correction, we obtained the long-flame coal outburst prediction (cutting desorption) index critical value. This value can provide a theoretical basis for outburst prediction and prevention of low rank coal mines and similar occurrence conditions of coal seams.

## Introduction

Coal is the primary energy source in China. Methane is not only a major coal mine product but also a valuable non-renewable energy resource [1]. However, methane emitted by coal mines from ventilation air is a significant greenhouse gas, which is likely to aggravate the global warming [2, 3, 4]. With increased mining depth, ground stress, and gas pressure, as well as low gas permeability of coal seams, the gas extraction became difficult before mining. The coal and gas outburst accidents are increasingly serious [5]. Of all coal mine gas accidents, outburst accidents have the highest occurrence and death toll in China in 2014 [6].

In coal seams, there is no protective seam, and we can only take regional outburst prevention measures of drainage gas by drilling crossing boreholes toward the coal seams. The floor rock roadway should be done in advance [7]. Coal seam gas pressure is an important regional outburst predictive index in evaluating the outburst danger level of coal seams. The coal cuttings desorption index, Δ*h*_2_, is one of the most important local outburst prediction index in forecasting the outburst risk of coal seams in China. The physical meaning of Δ*h*_2_ is the total gas desorption volume of coal samples (10 g) in the fourth and fifth minute after exposure to the atmosphere; coal samples are filtered from the cuttings falling from coal during drilling in underground coal mines [7].

To increase coal seam permeability and reduce the amount of extraction drilling and risk of outburst, researchers studied techniques and methods of hydraulics [8, 9, 10]. These methods include coal seam water injection, hydraulic punching, hydraulic cutting and high pressure hydraulic fracturing [11, 12, 13, 14].

Coal seam water injection is first applied to coal dust. Water injection has been adopted as mode of prevention and control of coal seam gas outburst by the former Soviet Union in the Donbas coalfield in 1946. In the late 1950s, almost all developed countries placed coal seam water injection into coal mining safety manuals [15]. The Shenyang Research Institute of China conducted test research of coal seam water injection on rock cross-cut coal uncovering a regional coal seam. They studied the effect of water injection on the coal seam, including the mechanism of coal seam water injection and process parameters [16, 17]. Other researchers conducted experiments of high-pressure abrasive jet slotting to increase coal seam permeability and improve gas extraction. Coal wall displacement was suggested as a test index of the use effect of hydraulic extrusion measures [18, 19]. The ways that hydraulic measures eliminate the outburst risk of coal seams are summarized as follows. Firstly, hydraulic measures increase the number of coal cracks and improve the permeability of coal seams, as well as the gas extraction effect [20, 21]. High pressure water will accelerate emissions of free gas and partly adsorbed gas [22, 23]. Secondly, water injected into coal fractures and pores closes the desorption channel of adsorption gas, and reduces the gas desorption speed [24, 25]. Thirdly, injected water makes the coal moist, improving the mechanical properties of the coal, making the stress concentration zone and increasing the width of the pressure relief protection belt. The essential outcomes of the various hydraulic measures are the increased water content in the coal and an alteration of the coal pore structure and fractures [26, 27]. In addition, the hydraulic measures will have a significant impact on the coal gas desorption speed, gas extraction and outburst prevention indicators of the coal seams [28, 29, 30].

At present, many studies on the hydraulic measures for coal seam outburst prevention and gas desorption kinetics are conducted in the field [31, 32, 33]. The former Soviet Union scholars conducted research on the gas desorption performance of water injection coal. Due to the limitation of experimental conditions, these researchers only injected high-pressure water to the adsorption of high pressure gas tanks in the laboratory. The coal samples were soaked in water, and desorption performance of each sample was subsequently measured; however, these laboratory measurements have differences from conditions in the field [34]. One of the technical difficulties is that adsorption high pressure gas in a special tank must be present before using the dry coal samples. Then, the high pressure atomized water can be injected into the tank, after the high pressure gas reaches adsorption equilibrium. Lastly, stirring and ultrasonic vibration uniformly distribute water in the coal sample, which helps test the gas desorption kinetics of the injected water in coals. Globally, low rank coals are responsible for approximately half of the world's total coal deposits. However, these low rank coals present a high moisture content, which significantly impacts their utilization processes, including lower power plant efficiency, increased transportation costs, higher CO_2_ emissions, and spontaneous combustion during storage [35].

Many institutions and engineering practices of the hydraulic measures were carried out both home and abroad in the outburst prevention of coal seams. However, there is a lack of in-depth theoretical and experimental research on gas desorption of low rank long-flame coal injected with water. This paper combines the high natural moisture content of Dalong long-flame coal as the research object, focusing on the influence of injected water on methane desorption dynamic characteristics and the outburst predictive index of coal. This study provides a theoretical basis for the prediction and prevention of outbursts of low rank coal mines and similar occurrence conditions of coal seams.

## Coal Sample

**Fig 1:**
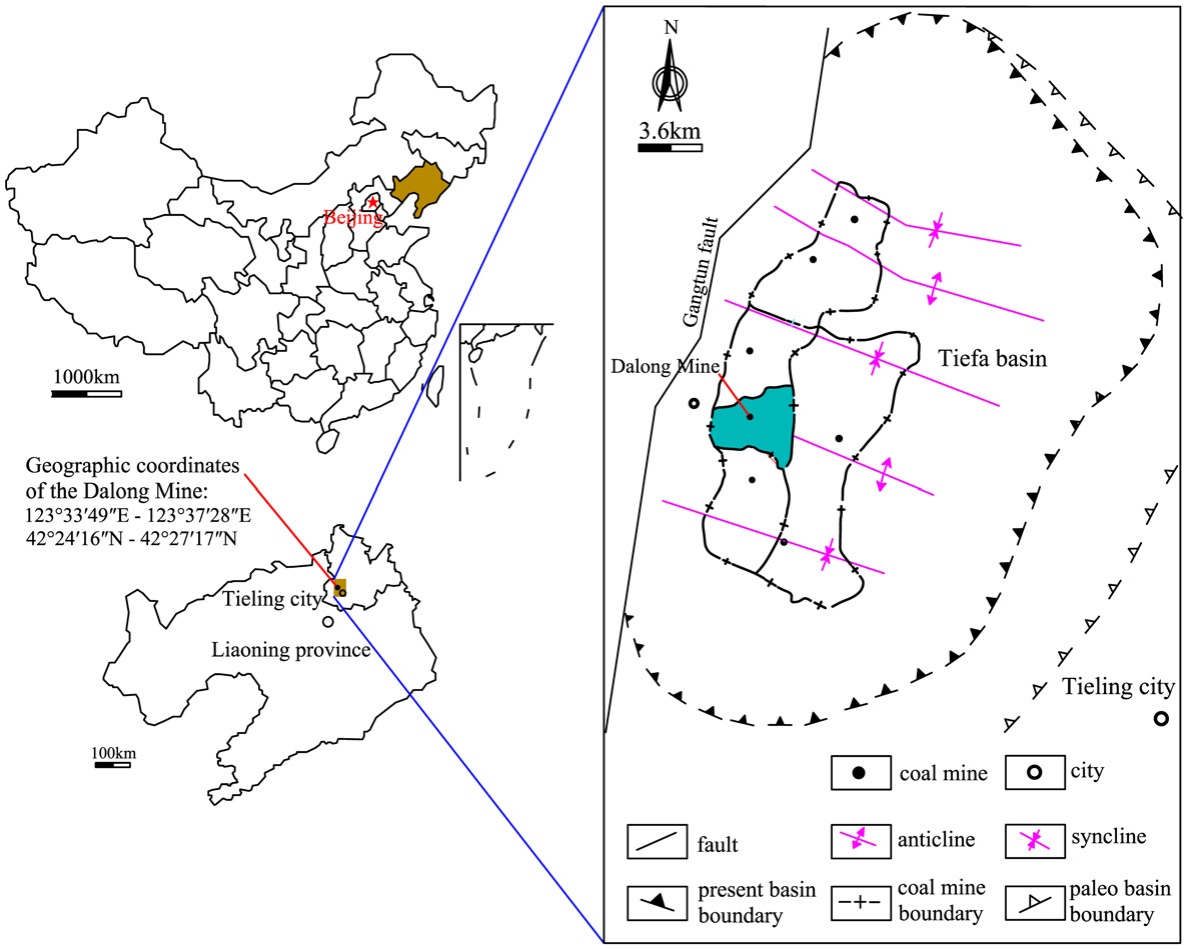
Map showing the study area (Dalong Mine) and a planar graph of the Tiefa coal basin.

Tiefa coal field is located in Tieling city in northeastern China's Liaoning province. The coal basin has a length of 29.5 km, a width of 17.4 km, and a total area of 513.3 km^2^. The Dalong Mine is located in the midwest of the Tiefa coal field (Fig 1), in which the coal bearing strata that are mainly used in mining belong to the Fuxin group of the Lower Cretaceous (K_1_f). The No. 13, 14, 15 and 16–1 coal seams are coal and gas outburst coal seams, with the average thickness of the No.13 coal seam of 2.74 m, burial depth of approximately 630 m, and gas pressure of 4.30 MPa. The experimental coal samples are all taken from coal seam No. 13 in the Dalong Mine.

## Experimental Method

We preprocessed the collection coal samples of the No.13 coal seam in the Dalong Mine (We state no specific permissions were required for these locations/activities. We confirm the field studies did not involve endangered or protected species). Firstly, we screened out coal sample particle sizes of 0.2–0.25 mm of approximately 100 g, and 1–3 mm coal samples of ~1000 g. Next, we placed the samples in a vacuum drying oven under a constant temperature of 100°C for 1 hour. Finally, the samples were stored in the ground in glass-stoppered flasks after cooling to room temperature. The basic information of the No.13 coal samples is shown in Table 1.

**Table 1:** Geochemical analyses of the coal samples in the Dalong Mine. Mois: moisture; Ash is on a dry basis; VM: volatile matter, on dry ash free (daf) basis; FC = fixed carbon (daf basis); R_o_: random vitrinite reflectance.

Sample	Coal seam No.	Mois (wt.%)	Ash (wt.%)	VM (wt.%)	FC (wt.%)	R_o_ (%)
DL#131	13	6.84	13.75	39.44	39.97	0.57
DL#132	13	7.16	15.23	40.91	36.70	0.59

The average moisture content of the coal samples DL#131 and DL#132 is 7.0%. The vitrinite reflectance (R_o_) of the samples is 0.58% (Table 1). Coal rank belongs to low rank bituminous C according to international standard (ISO11760) [36].

### Adsorption Experiments

Coal samples of 50 g and 0.2–0.25 mm were prepared in an adsorption tank. The water bath temperature was set at 60°C. The vacuum pump was opened, and vacuum degassing was performed until the vacuum gauge displayed a pressure of 4 Pa. Next, the vacuum pump valve and the tank valve were closed. The water bath was then set to a constant temperature of 30°C. The gas adsorption volumes were tested under equilibrium pressures of 1 MPa, 2 MPa, 3 MPa, 4 MPa and 5 MPa. The volume of the adsorbed gas was collected by using the drainage gas-collecting method.

### Desorption Experiments

Desorption test was conducted as follows. Firstly, the prepared samples of 50 g and particle sizes of 1–3 mm were placed in a tank. The water bath temperature was set at 60°C. The vacuum pump was opened, and vacuum degassing was performed for two hours. Next, the water bath was set to a constant temperature of 30°C. The high pressure gas cylinder was filled and the tank was connected as the samples reached adsorption equilibrium. The equilibrium pressures of this test were 4.30 MPa, 3.50 MPa, 3.18 MPa, 2.19 MPa and 1.16 MPa. Pressure is released instantaneously after the sample reaches adsorption equilibrium. The gas desorption process is described by the calculation of the amounts of gas desorption at different desorption times.

### Methane Desorption Test of Coal with Injected Water

A test device has been developed which is called the methane desorption kinetics characteristics test of coals of water injection under high pressure-[37]. The desorption characteristics test of the samples injection of different moisture was conducted in the laboratory. Steps of the experiment are as follows. Firstly, 60 g dry coal samples were place into the sample tank and kept in an oil bath at a constant temperature of 60°C, as degassing began. Secondly, the constant temperature of the oil bath was reduced to 30°C. The coal sample tank was filled with methane and we affirmed that the samples reached adsorption equilibrium. Thirdly, the mixing device injected water into the sample tank and stirring occurred for 30 min; the coal sample was then allowed to cool and reach adsorption equilibrium again. The coal sample tank and the valve of desorption measuring device were opened to release free gas into the gas sample bag. The tee was rotated and the timer was started when the pressure in the sample tank dropped to 0 MPa. The desorbed gas was allowed to flow into the desorption instrument, as the cumulative desorption volume was recorded in the measuring cylinder each minute. The testing continued until no desorption occurred under the condition of atmospheric pressure. Finally, the tank was opened and the samples were separated from three layers of the sample for proximate analysis. The average moisture content of the three collected samples was collected as an additional moisture value.

### Determination Methods of the Coal Cuttings Desorption Index Δ*h*_2_

**Fig 2:**
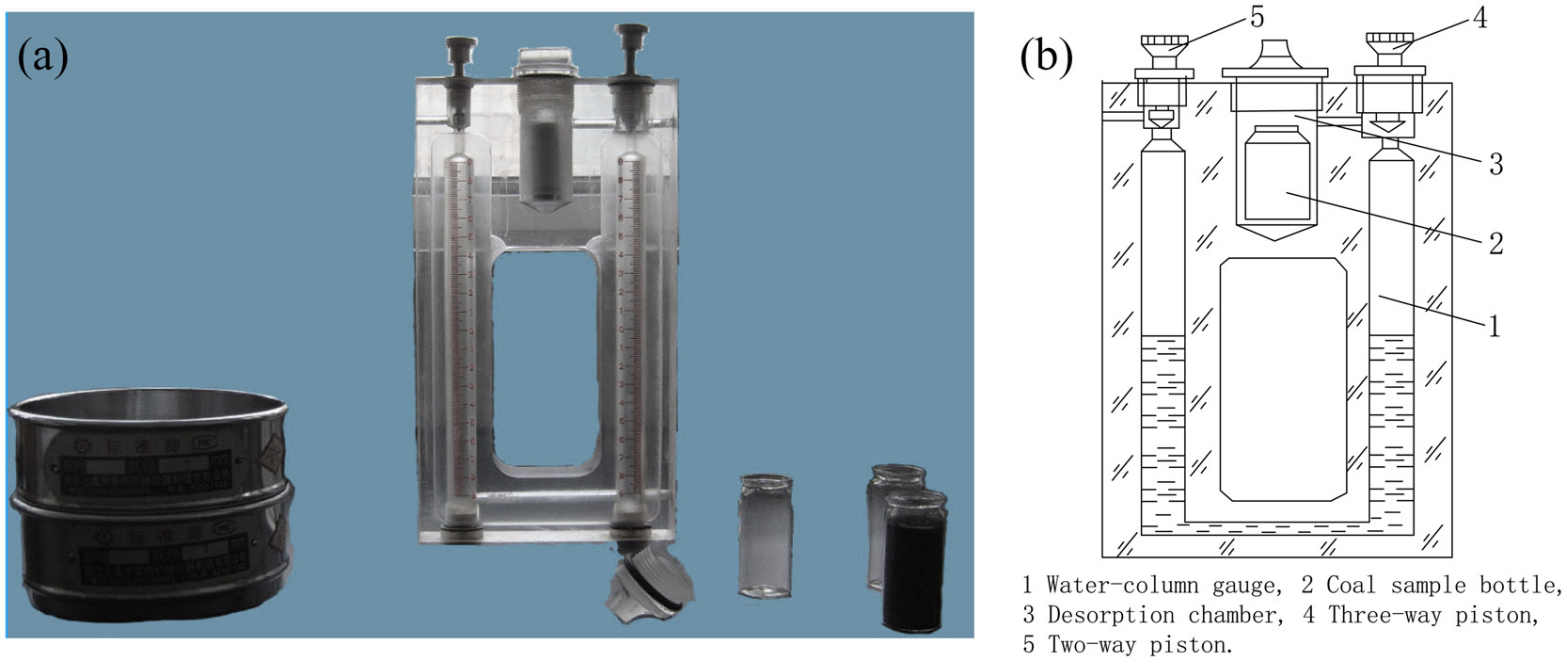
Picture of real products (a) and structure schematic (b) of the coal cuttings gas desorption test instrument (MD-2).

The MD-2 methane desorption instrument (Fig 2), which is produced by the Coal science Institute of Fushun, was used to measure the coal cuttings desorption index (Δ*h*_2_). The basic principle of the instrument is that use of the residual gas pressure slacked in coal cuttings releases gas into a confined space [38]. The volume and pressure are changed under the conditions of no degassing and charging gas. The form of the pressure difference of water-column gauge was used to represent the quantity of gas desorbed from coal cuttings. The unit of Δ*h*_2_ is Pa. For the gas desorption instrument, there is a relation between the gas desorption volumes (*Q*) of the fourth and fifth minute per gram of sample and Δ*h*_2_, as shown in Eq (1).

$$Q=0.0083\times \Delta h_{2}÷10$$ (1)

where *Q* stands for the gas desorption volume, in cm^3^/g. 10 is the weight of coal samples, g. The constant 0.0083 is the structure constant of the gas desorption instrument (MD-2).

## Results and Discussion

### Adsorption Characteristic of Long-Flame Coal

Gas adsorption of coal is a type of physical absorption [18]. The adsorption characteristic of coal is usually represented by the adsorption isotherm, which is a type of special curve that shows the variation of gas adsorption volume change with the gas pressure. Research shows that when coal adsorbs methane, the adsorption isotherm is accordance with the Langmuir equations [39]:

**Fig 3:**
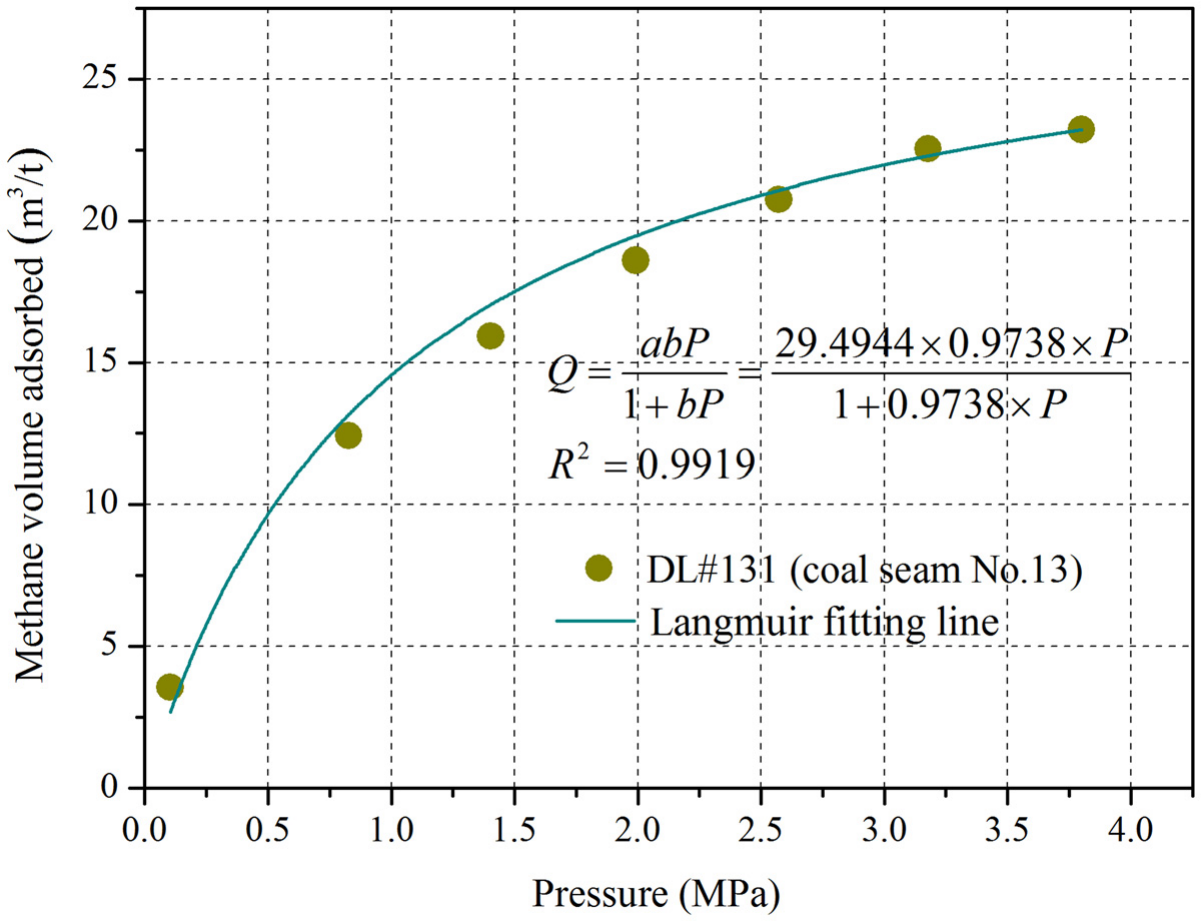
Relationship between the methane adsorption amount and gas pressures of the No.13 coal seam.

The adsorption isotherm of the Dalong No.13 coal seam is shown in Fig 3. With the increase of gas pressure, the quantity of gas adsorption also increases. However, the growth rate of the adsorption volume decreases gradually. When the gas pressure increases indefinitely, the quantity of the coal gas adsorption tends to a limiting value. The Langmuir volume (adsorption constant *a*) of the No.13 coal seam is 40.26 m^3^/t. The Langmuir volumes of different coal seams in Chinese coal fields cover a broad range. The Langmuir volume usually ranges between 13 and 60 m^3^/t (dry and ash free) and the adsorption constant ***b*** usually ranges between 0.4 and 2.0 MPa^-1^[18].

### Desorption Kinetics Characteristics of Long-Flame Coal

**Fig 4:**
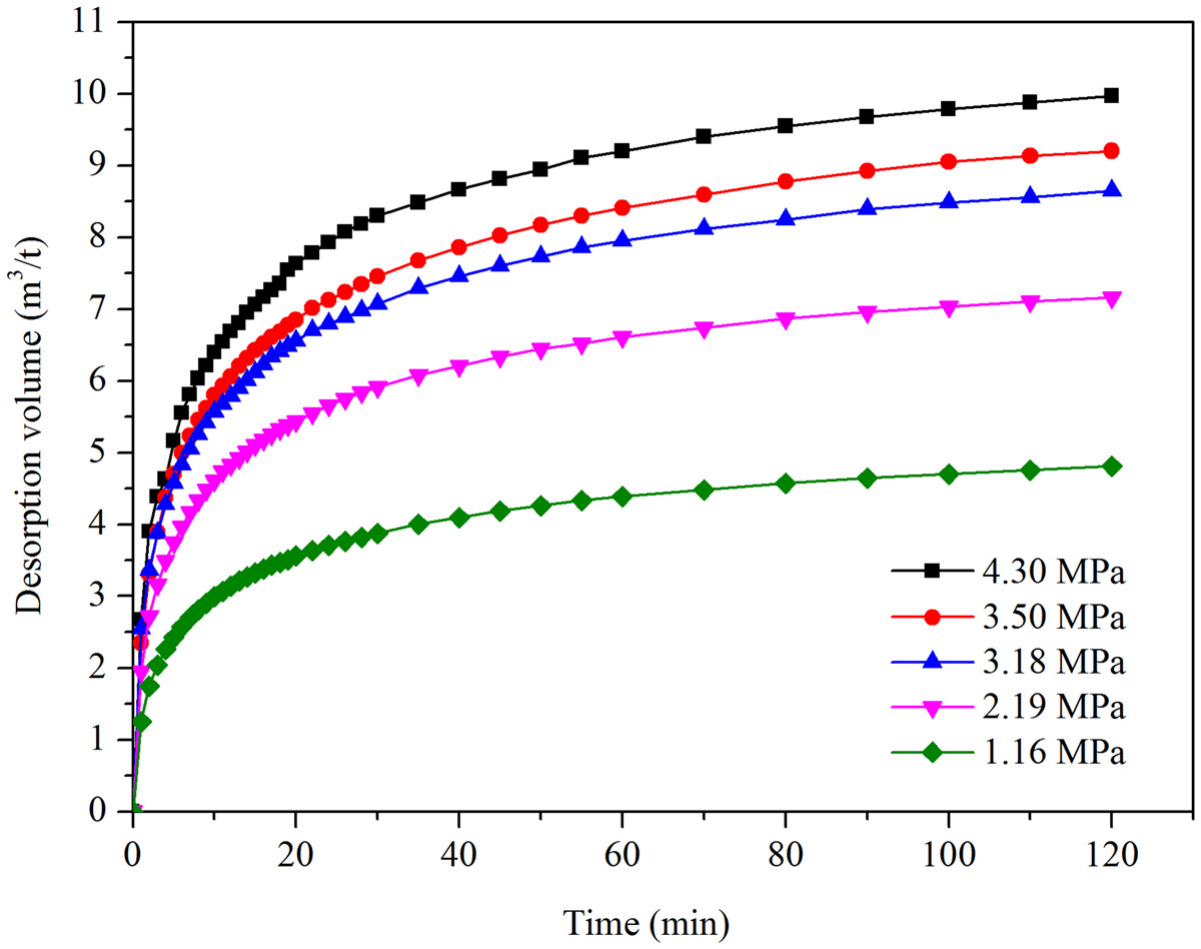
Relation between gas desorption volume and time under different pressures in the No. 13 coal seam.

The relationship between the desorption amount and desorption time under different pressures of the Dalong samples is shown in Fig 4.

Fig 4 indicates that the quantity of gas desorption is much greater in the first 30 min. However, the quantity of desorption gas increases gradually between 80 to 120 min. As time increases, the relationship between the gas desorption volumes and gas pressures conform to the Langmuir adsorption isotherm. When the gas pressure of the adsorption equilibrium is 4.30 MPa, 10.1 mL/g of methane was desorbed in 120 min. When the gas pressure decreased to 1.16 MPa, 4.6 mL/g methane was desorbed in 120 min (Fig 4). The gas desorption curves of high pressure are always located above the curves of low pressure at different desorption times. The desorption curve gradient of high pressure is initially larger than that of low pressure, which means the initial desorption speed is much higher under the condition of high pressure. If the pressure of adsorption equilibrium is much higher, the quantity of gas desorption will simultaneously be considerably higher.

### Influence of Injected Water on the Gas Desorption Characteristics of Coal

The moisture in coal exists in a variety of states and chemical combinations, including adsorbed, capillary and free states [40]. The adsorption moisture plays a more important role in adsorption/desorption ability-[41]. At low pressure, water adsorption is strongly influenced by the presence of functional hydrophilic groups. These oxygen-containing groups are able to act as primary adsorption centers due to the formation of hydrogen bonds with adsorbing water molecules [31, 32].

When there is some moisture in the coal, the quantity of methane adsorption will be reduced significantly because the adsorption ability of coal for water vapor is larger than for methane. At present the determination of the coal adsorption isotherm is usually conducted for dry coal samples. There are numerous empirical formulas demonstrating the influence of moisture in coal in terms of the quantity of gas adsorption. The Aegean empirical formula is used most widely [18]:

$$X_{w}=\frac{X_{d}}{1+0.31W}$$ (2)

where *X*_*W*_ is the gas adsorption amount of wet coal, m^3^/t. *X*_*d*_ is the methane adsorption amount of dry coal, m^3^/t. *W* is the moisture content of coal, %.

Eq (3) does not consider the influence of moisture on gas adsorption of coals having differences in metamorphic degree. We should note that empirical formulas that consider the influences of moisture on gas adsorption volumes all have large deviations. Because the moisture is determined according to the testing standard, it can be in different states, which can have a wide range of effects on gas adsorption. The chemical bonding of water to coal or minerals contained in coal has no influence on the amount of gas adsorption. The bound water in the adsorption state in micropores plays a vital role in the process of reducing the adsorption capacity of coals. Capillary water and free water that exist in large pores and capillaries can dissolve little methane, but have no significant effect on gas adsorption. There will inevitably be large deviations when we use the empirical formula for the moisture content in coal to determine the quantity of gas adsorption because there is no separate method at present to measure the quantity of absorbed water in coal. To measure gas adsorption quantity of coal more accurately, we should do quantitative study of gas adsorption properties of coal injected by water in the laboratory.

**Fig 5:**
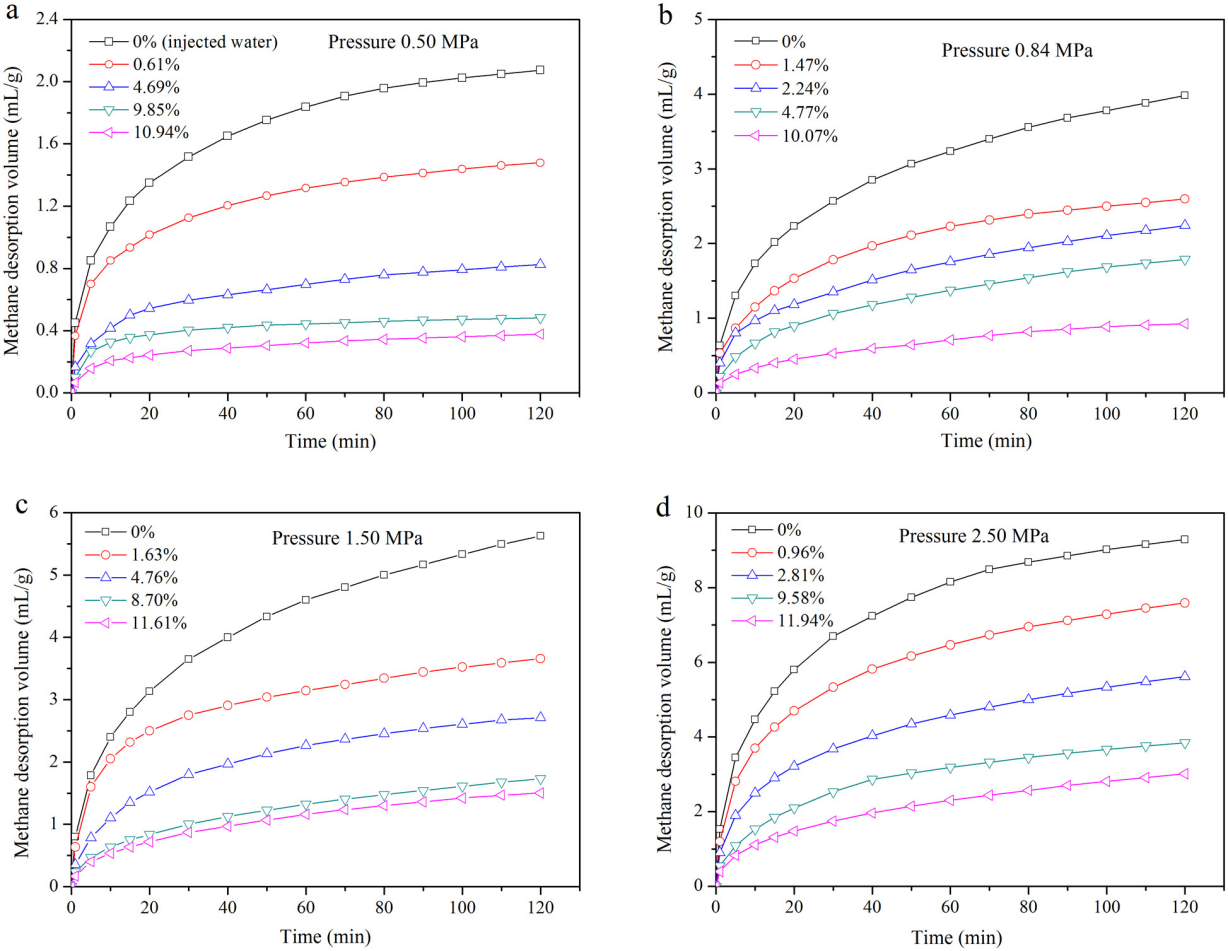
Relationship between gas desorption volumes and time under the condition of different pressures and water injection.

The coal in the Dalong Mine has a low degree of metamorphism. The coal in this mine is a type of soft coal with high natural moisture content. Studies show that the lower the metamorphic grade of coal, the higher the coal’s natural moisture content is. The natural moisture content of lignite can be as high as 20%, which is associated with the internal rigid frame structure of lignite. We performed experiments on the gas desorption quantity changes with desorption time under the conditions of different pressures and water injection rates. The gas equilibrium pressures were 0.50 MPa, 0.84 MPa, 1.50 MPa and 2.50 MPa. The relationship between gas desorption volumes and desorption time under the condition of different gas pressures and water injection are shown in Fig 5.

With the increase of injected water content of coal, the gas desorption volume decreased under the same pressure conditions (Fig 5). The higher the injected water content is, the lower the amount of accumulated desorption is after 120 min. At the first 20 min, the gas desorption speed was fast. However, between 100 min and 120 min, the gas desorption volume growth speed became slow. When the moisture content was 0%, as the adsorption equilibrium pressure increased from 0.5 MPa to 2.5 MPa, the gas desorption amount of the coal sample increased from 2.2 mL/g to 9.3 mL/g in 120 min (Fig 5a). When the pressure was set at 1.50 MPa, the moisture content increased from 0% to 0.96%, and the gas desorption amount decreased from 5.7 mL/g to 3.6 mL/g, which reduced for 36.8% (Fig 5c). As the moisture content increased from 8.70% to 11.61%, the amount of gas desorption decreased from 1.5 mL/g to 1.3 mL/g in 120 minutes, which reduced by 13.3% (Fig 5c). Field experiments of the coal seam water injection in the Dalong Mine indicate that when the moisture content increased to a certain value (critical moisture content), the gas desorption quantity would change little or no longer desorbs. CH_4_ desorption capacity of coal was reduced by ~1.8 kgt^−1^ for each 1% increase in moisture [42]. There is a limit to hydraulic curriculum and it is inappropriate to increase the moisture content of coal blindly. To prevent gas outbursts, as well as preserve water resources, we should optimize coal water injection parameters.

#### 4.4 Effect of injected water on the coal cuttings desorption index

Based on the outburst statistical data analysis of 26 gas outburst coal mines in China, the relationship between the minimum outburst pressure and the volatile matter of coal was used to calculate the coal firmness coefficient, in accordance with the following equation [18]:

$$P_{\text{min}}=A(0.1+B\times VM\times f)$$ (3)

where *P*_min_ is the minimum outburst pressure of a coal seam, MPa. VM is the volatile matter, on daf basis, wt.%. *f* is the firmness coefficient of the soft layer in coal. *A* is a constant (*A* = 5 under these statistical conditions). *B* is a constant (*B* = 0.017 under these statistical conditions).

**Fig 6:**
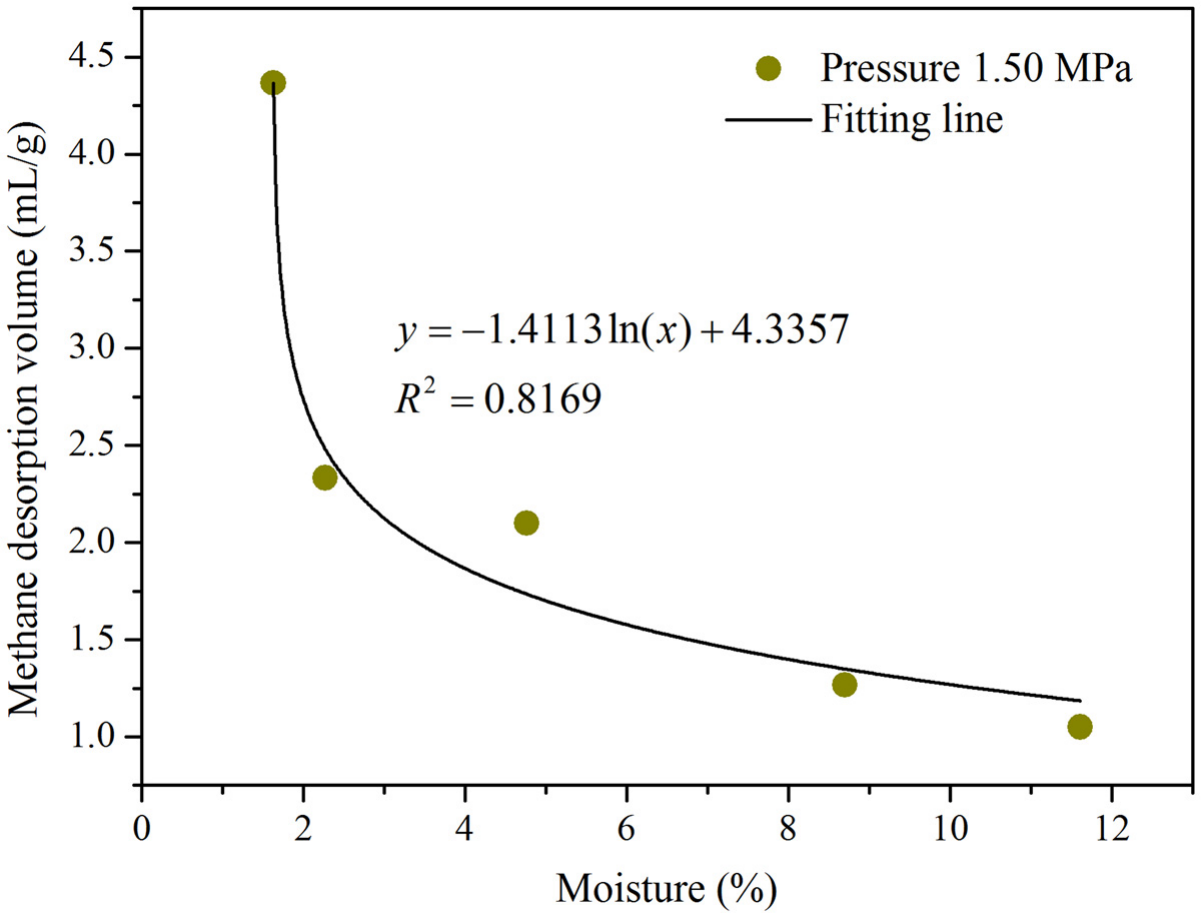
Relationship between the desorption quantity of the 4th and 5th min and injected water under 1.50 MPa.

According to Eq (3), the measured firmness coefficient of soft layer in coal (*f* = 0.50) and volatile matter (VM = 40.18) of the No.13 coal seam in Dalong Mine are shown in Table 1. The minimum outburst pressure of the No.13 coal seam is ~2.20 MPa. Considering the general threshold of the regional outburst predictive index (gas pressure 0.74 MPa) and a certain safety coefficient [7]. The gas pressure value 1.50 MPa is chosen as the critical value of the gas pressure sensitive index. There is a need to test the gas desorption data corresponding to the coal cuttings predictive index Δ*h*_2_ under the condition of critical gas pressure 1.50 MPa. According to the definition of the coal drill cuttings index Δ*h*_2_ in the introduction section of this paper, the index corresponds to the initial gas desorption quantity. The gas desorption quantity of the 4th and 5th minutes of different injected water contents under a pressure of 1.50 MPa are shown in Table 2. Fitting of the desorption quantities of the 4th and 5th minutes and different injected water contents under pressure of 1.50 MPa can obtain the relation curve shown in Fig 6.

**Table 2:** Desorption quantity of the 4th and 5th min of injected water content under a pressure of 1.50 MPa.

Moisture (%)	1.63	2.27	4.76	8.70	11.61
desorption volume in the 4th and 5th min (mL/g)	4.37	2.33	2.10	1.27	1.05

The gas desorption quantity in the 4th and 5th minutes relation with moisture content conforms to a logarithmic relationship. The following formula is used as Eq (4):

y=−1.4113ln(x)+4.3357 (4)

where *y* is the gas desorption volume in the 4th and 5th min, mL/g. x is the injected water content, %.

Before testing of the drilling cuttings index in the laboratory, the coal sample needs to be vacuumed, which causes water loss in the coal. Proximate analysis showed the moisture of the vacuumed coal sample is only ~0.5% after the experiment. Therefore, we can obtain the correction coefficient of the moisture content of the corresponding desorption amount if we know the corresponding desorption amount under different moisture contents and a moisture content of 0.5%. The relationship is the basis of the following Eq (5):

Ax=yxy0.5 (5)

Where *A*_*x*_ is the correction coefficient when coal sample moisture content is x%. *y*_*x*_ is the gas desorption quantity when moisture content is x%, mL/g.*y*_0.5_ is the gas desorption amount when the moisture content is 0.5%, mL/g.

**Fig 7:**
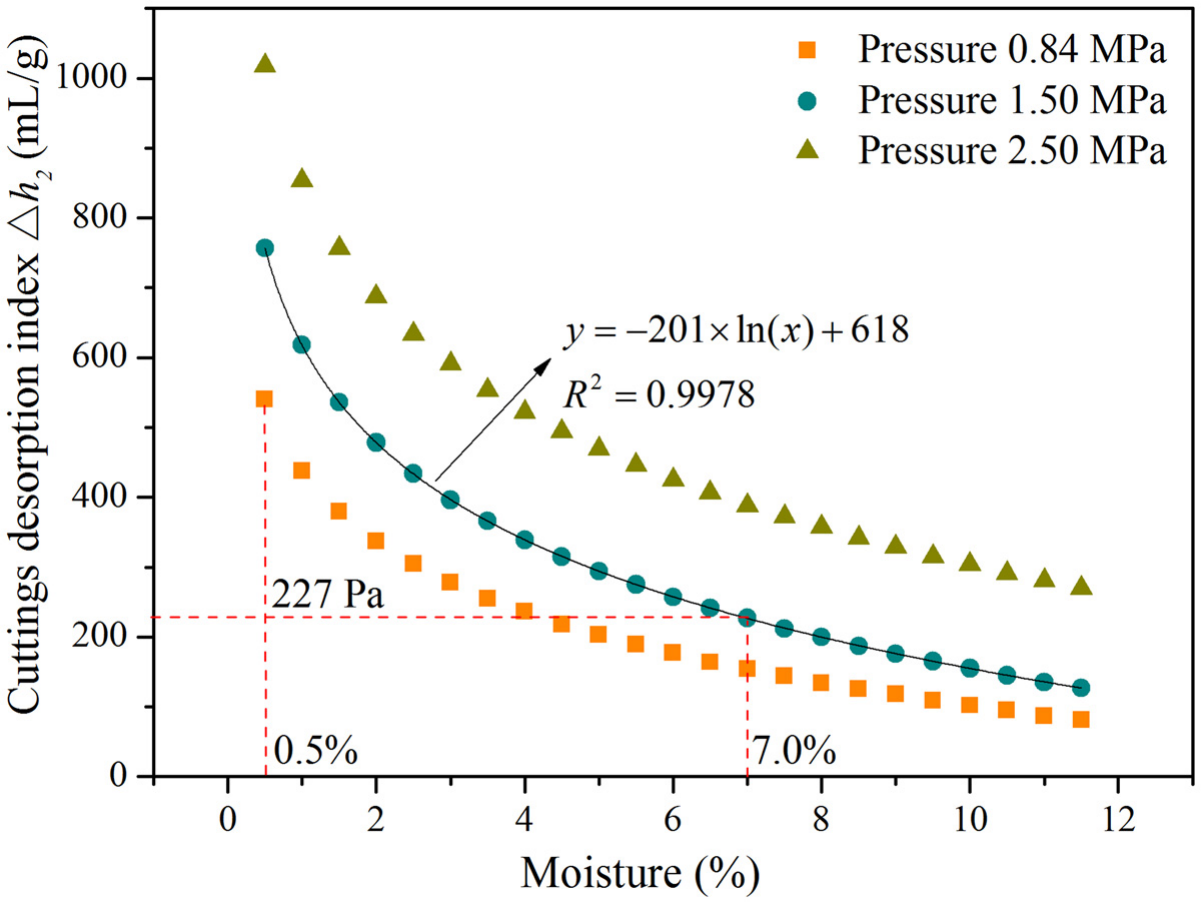
Relationship between coal cuttings desorption index Δ*h*_2_ and moisture of the No.13 coal seam.

The laboratory tests indicate the coal cuttings desorption indexes Δ*h*_2_ of the dry coal samples are 415 Pa, 582 Pa and 783 Pa, under the pressures of 0.84 MPa, 1.50 MPa and 2.50 MPa, respectively. The Δ*h*_2_ values are 540 Pa, 757 Pa and 1018 Pa, respectively, after multiplied the reserved safety factor of 1.3. The relationship between the outburst prediction index Δ*h*_2_ and moisture, after considering the impact of moisture correction, is shown in Fig 7.

As shown in Fig 7, the data fit indicates a logarithmic relationship between the coal cuttings desorption index Δ*h*_2_ and moisture content. The moisture content of the measured DL#131 and DL#132 coal sample are 6.84% and 7.16%, respectively, with an average value of 7.0% (Table 1). A moisture content of 7.0% corresponds to the Δ*h*_2_ value of 227 Pa. Thus, the value 227 Pa was set for the outburst prediction sensitivity index Δ*h*_2_ of the No.13 coal seam in the Dalong Mine. The quantitative case studies on the effect of injected water on the gas desorption of long-flame coal in the Dalong Mine could provide a good example for other gas coal mines with low rank coals.

## Conclusions

1. The Dalong coal is composed of low rank coals with an R_o_ value of 0.57% to 0.59%. The natural moisture content of coal samples is higher with an average moisture value of 7.0%. The effect of water on the gas adsorption of coal is mainly that water molecules block the gas flow channel, and the competition of adsorption between methane and water molecules occupies part of the adsorption potential in the coal matrix. The adsorption test showed that the Langmuir volume of the Dalong coal sample is ~40.26 m^3^/t, indicating a strong gas adsorption ability.
2. The desorption test indicates that the gas desorption quality is considerably greater in the first 30 min. However, the desorption volume increased gradually in the last 80 to 120 min. When the gas pressure of adsorption equilibrium is 4.30 MPa, 10.1 mL/g methane was desorbed in 120 min. When the gas pressure decreased to 1.16 MPa, 4.6 mL/g gas was desorbed in 120 min.
3. Experiments of the impact of external moisture on gas desorption indicate that the gas desorption volumes and moisture content conform to a logarithmic relation. The higher water content (lower than the critical saturation of water content), the smaller the accumulation desorption volume. After moisture correction, we can obtain the outburst prediction (coal cuttings desorption index) index critical value of long-flame coal. This index can provide a theoretical basis for the low rank coal mines to determine the critical value of the coal seam outburst prediction index.
